# Effects of Hot Pixels on Pixel Performance on Backside Illuminated Complementary Metal Oxide Semiconductor (CMOS) Image Sensors

**DOI:** 10.3390/s23136159

**Published:** 2023-07-05

**Authors:** Bingkai Liu, Yudong Li, Lin Wen, Xiang Zhang, Qi Guo

**Affiliations:** 1 Key Laboratory of Functional Materials and Devices for Special Environments, Xinjiang Technical Institute of Physics and Chemistry, Chinese Academy of Sciences, Urumqi 830011, China; 2 Xinjiang Key Laboratory of Electronic Information Material and Device, Urumqi 830011, China; 3 Aerospace System Engineering Shanghai, Shanghai 201109, China

**Keywords:** backside illuminated CMOS image sensors, hot pixels, displacement damage effects, proton irradiation, random telegraph signal

## Abstract

Effects of hot pixels on pixel performance in light and dark environments have been investigated in pinned photodiode 0.18 μm backside illuminated CMOS image sensors irradiated by 10 MeV protons. After exposure to protons, hot pixels and normal pixels are selected from the whole pixel array, and their influences on key parameters are analyzed. Experimental results show that radiation-induced hot pixels have a significant impact on pixel performance in dark environments, such as dark signal nonuniformity, long integration time, and random telegraph signal. Hot pixels are caused by defects with complex structures, i.e., cluster defects. Furthermore, the dark current activation energy result confirms that the defects causing the hot pixels have defect energy levels close to mid-gap.

## 1. Introduction

Backside illuminated CMOS image sensors (BSI CIS) feature a higher sensitivity compared to the frontside illuminated CIS, and have been extensively used for space imaging missions [1,2,3]. The space-borne BSI CIS suffers from displacement damage effects due to the existence of high-energy protons in space environments. The elemental process of displacement damage is the dislodging of the atoms from their normal lattice sites due to the impinging of energetic particles, and the resulting influence of displacement damage causes the performance degradation of devices through the introduction of defect energy levels within the Si bandgap. For the BSI CIS, displacement damage leads to the production of hot pixels. Hot pixels refer to the anomalous pixel with high dark current compared to the normal pixels with moderate dark current increase after irradiation, and have a significant influence on space imaging missions [4,5,6], especially for dim target detection missions.

Much effort has been made to study displacement damage-induced hot pixels [7,8,9,10], and three possible physical mechanisms responsible for the production of hot pixels have been proposed: (a) electric field enhancement (EFE) effect: the thermal generation rate of the defects is significantly enhanced through Poole−Frenkel effect or phonon-assisted tunning when high electric field (~105 V/cm) exists within the pixel due to unreasonable doping processes or device structures [11]; (b) intercenter transfer mechanism: two adjacent divacancies with different charge state can interact, and the generation rate can be improved in a significant way, that is, exchange reaction ($V_{2}^{0}+V_{2}^{0}\to V_{2}^{+}+V_{2}^{-}$). Moreover, divacancies are common defects in the displacement damage [12]; (c) the defects closer to the mid-gap have a higher generation rate than the defects away from the mid-gap, according to the Shockley-Read-Hall (SRH) theory [13]. 

Although these mechanisms have been proposed in previous work, it can be known that the generation of hot pixels depends on many factors, such as the electric field in the pixel, the defect types and density, and the defect energy level within the Si bandgap. Hence, displacement damage-induced hot pixel is still a complicated issue, and the dominant mechanism needs to be identified. Moreover, the previously published papers mainly focus on the behaviors of hot pixels. Additional work is needed to investigate the effects of hot pixels on pixel performance in light and dark environments. 

The purpose of this paper is to investigate the effects of hot pixels on pixel performance in light and dark environments and to provide a new perspective for understanding hot pixels. We randomly select the hot pixels and normal pixels from the whole array, and study their influences on key parameters, including full well capacity, quantum efficiency (QE), conversion gain (CVG), dark signal nonuniformity, and random telegraph signal (RTS). From the perspective of the defects, the correlation between the hot pixels and RTS pixels is discussed in detail. In addition, the physical mechanism of radiation-induced hot pixels is identified through dark current activation energy measurement.

## 2. Experiment Details

### 2.1. Device Information

**Figure 1:**
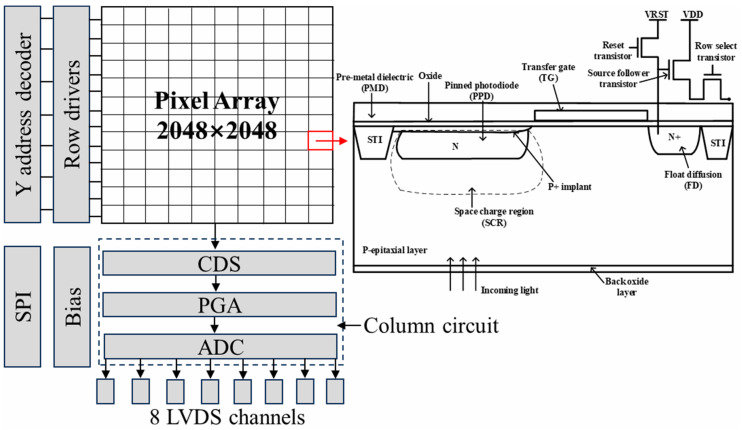
Block diagram of the studied BSI CIS and the cross-section of a pixel using the PPD scheme.

**Figure 2:**
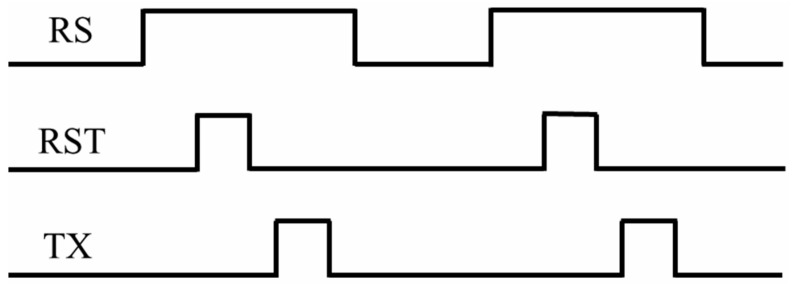
The timing sequence of 4T pixels.

**Figure 3:**
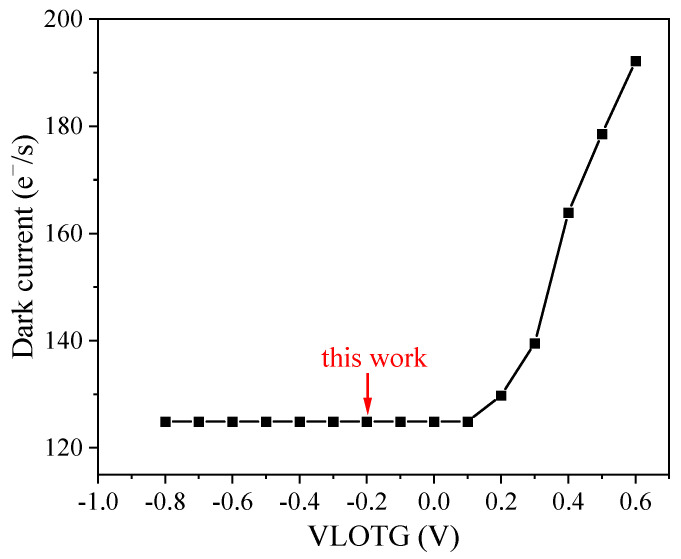
Dark current as a function of VLOTG before proton irradiation.

The devices under study are the BSI CIS manufactured in a 0.18 μm CMOS process dedicated to imaging. The image sensors are constituted of 2048 × 2048-11 μm-pitch-4T pixels using the pinned photodiode (PPD). The image sensor supports two modes: STD mode at 48 fps and HDR mode, which is designed to achieve a high dynamic range (93 dB) at 24 fps. The HDR mode is selected to acquire raw images in this work. The shutter type of the image sensor is an electronic rolling shutter. The temporal dark current is 1.6 e^−^, and the full well capacity is 90 ke^−^. Figure 1 depicts the block diagram of the studied BSI CIS and the cross-section of a pixel using the PPD scheme. The device integrates a column readout circuit, a temperature sensor, a phase-locked loop (PLL), a serial peripheral interface (SPI), and 8 low-voltage differential signalings (LVDS) output pairs on-chip. The power consumption of the BSI CIS is less than 650 mW at full speed mode. The column readout circuit contains the correlated double sampling (CDS), the programmable gain amplifier (PGA), and a 12-bit column-parallel analog-to-digital converter (ADC) for eliminating reset noise and fixed pattern noise in pixel, amplifying the effective signal and reducing equivalent input noise, and converting the analog signal to digital signal. It can also be seen from Figure 1 that a pixel cell consists of a PPD and four control transistors, including a transfer transistor, source follower transistor, reset transistor, and row select transistor. In a deep submicron process, shallow trench isolation (STI) structure is used to achieve electrical and optical isolation between pixels. The sensitive region of displacement damage is the space charge region (see the dotted line in Figure 1), in which the radiation-induced defects are active defects, and the active defects play a crucial role in parameter degradation. Figure 2 shows the timing sequence of 4T pixels. Firstly, a row-select pulse (RS) is enabled, and the specific row can be accessed. Then, a reset pulse (RST) is enabled to reset the float diffusion to a fixed voltage level, and the first readout of the float diffusion voltage is stored in a capacitor of the CDS circuit. A pulse (TX) is applied on the transfer gate, and the photo-generated electrons are transferred from the PPD to the float diffusion. Now the second readout of float diffusion voltage is also stored in another capacitor of the CDS circuit. Finally, the effective signal can be obtained through the processing of the CDS circuit and equals the difference between two float diffusion voltages. VLOTG refers to the transfer gate voltage during integration and is an important parameter influencing dark current and full well capacity [14]. Figure 3 depicts dark current as a function of VLOTG before proton irradiation. When the VLOTG is below 0.1 V, the transfer gate channel is in the accumulation regime, and no apparent dark current increase is observed. Above 0.1 V, the transfer gate channel is depleted, and the dark current significantly increases due to the contribution of the interface states. In this work, this voltage is set to −0.2 V, and total ionizing dose effects caused by proton irradiation can be effectively mitigated.

### 2.2. Irradiation and Measurement

BSI CIS was irradiated by 10 MeV protons at the Institute of Heavy Oon Physics, Peking University, CHN. The proton fluence is 5 × 10^9^ p·cm^−2,^ and the irradiation duration is 100 s. The cumulative displacement damage dose is 39.4 TeV/g, and the total ionizing dose is 2.8 krad(Si). The proton flux is 5 × 10^7^ p·cm^−2^·s^−1,^ and the proton beam homogeneity is within ±5%. The image sensors were irradiated at room temperature with all the pins grounded.

**Figure 4:**
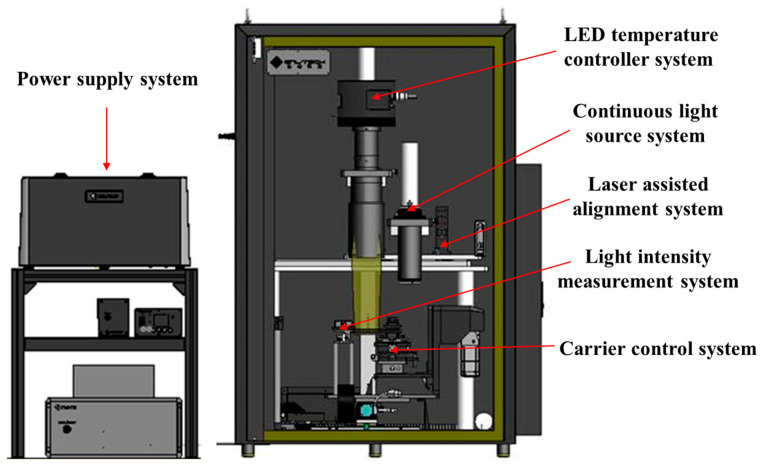
Schematic diagram of QE measurement apparatus.

Key parameters test and characterization were performed at 24 °C one week after exposure to protons according to the standard for characterization of image sensors and cameras (EMVA1288). RTS pixel test was carried out with the successive acquisition of the raw images. The duration of raw image acquisition is 3 h, and the integration time is set to 1 s. RTS pixels can be detected from the subarray of the interest by an automated RTS test software, which is implemented using MATLAB software and is based on the sharp edge detection technique described in ref. [15]. The RTS software can provide the characteristics of the RTS pixels, such as transition amplitude, discrete levels, time constant, RTS pixel count, and RTS pixel locations. To obtain the activation energy of the defects, the dark current test was performed with temperatures ranging from 0 °C to 24 °C. Considering the double sampling temperature of dark current (about 7 °C in the studied devices), four temperature points are selected (including 0 °C, 7 °C, 14 °C and 24 °C). The measurement of the QE is a little different from the test setup of the other parameters. Figure 4 shows the schematic diagram of the QE measurement apparatus. The measurement apparatus contains a continuous light source system that can generate a desired wavelength ranging from 200 nm to 1000 nm, and a light intensity measurement system for the calculation of photon count in the pixel. The light uniformity of the measurement apparatus is greater than 99%, and the light stability is less than 1%.

## 3. Results and Discussion

### 3.1. Hot Pixel Selection

**Figure 5:**
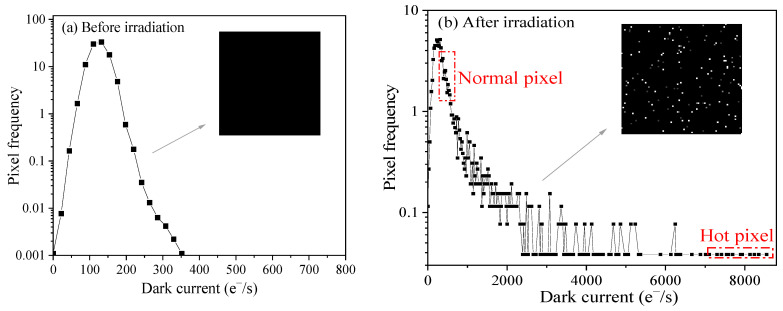
Dark current distributions (**a**) before irradiation and (**b**) after 10 MeV proton irradiation.

Figure 5 shows dark current distributions before and after irradiation. It can be seen that the dark current distribution is the Gaussian distribution, and the captured image is uniform before irradiation (see Figure 5a). After exposure to protons, dark current distribution consists of a Gaussian peak and exponential tail. The captured image is nonuniform, and numerous “bright spots” can be observed (see Figure 5b). Interaction types for the protons include the inelastic collision with atomic electrons, Coulomb elastic scattering, and elastic and inelastic nuclear scattering with the nucleus. Except for the inelastic collision with electrons, the remaining three interactions can give rise to the production of displacement damage. The characteristics of Coulomb elastic scattering are a big cross-section and low damage energy transmitted to the Si atoms, and this interaction impacts all the pixels (corresponding to the Gaussian peak of the dark current distribution). The elastic and inelastic nuclear scattering have small cross sections and high damage energy, and impact a part of pixels (corresponding to the exponential tail of the dark current distribution). Inelastic nuclear scattering has a smaller cross-section and higher damage energy compared to elastic nuclear scattering. It has been reported that the dark current linearly increases with displacement damage dose, and the proportionality coefficient is defined as the dark current universal factor (K_dark_), which is independent of the particle types and energy [16,17]. Thus, the dark current increase due to inelastic nuclear scattering is larger than that of elastic nuclear scattering since higher damage energy is transmitted to the displacement damage process.

Hot pixels are located at the exponential tail, and normal pixels are in the Gaussian peak. According to the definition of the hot pixels, we select 10 hot pixels with the maximum dark current from the exponential tail and 10 normal pixels from the Gaussian peak. The normal pixel is randomly selected from the whole array and meets statistical requirements. Table 1 summarizes the information on the selected hot pixels and normal pixels. It can be seen the dark current of hot pixels is much larger than that of normal pixels (the mean dark current of hot pixels is 7956.1 e^−^/s, and the one is 415.4 e^−^/s for normal pixels). In order to obtain the response of the selected pixels in light and dark environments, the locations of the selected hot pixels and normal pixels are recorded to track these pixels.

### 3.2. Pixel Performance at Light Environments

**Figure 6:**
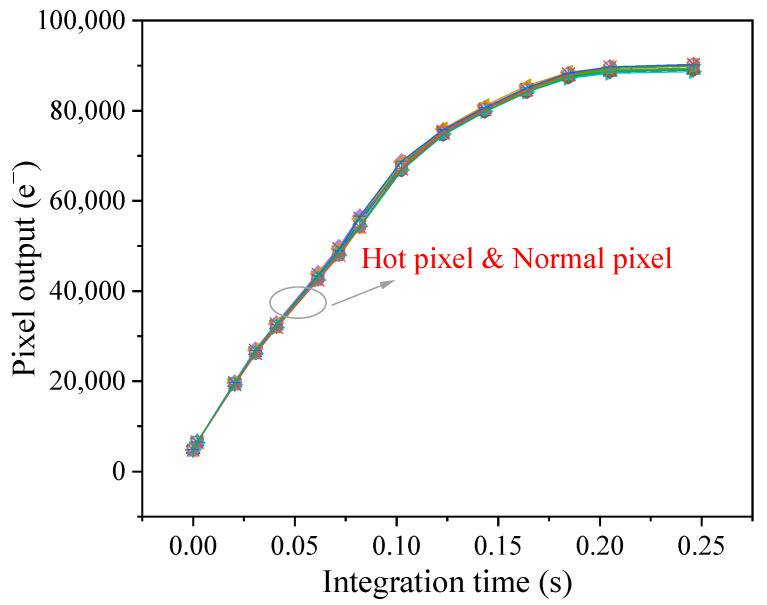
Pixel output as a function of integration time under light environments.

Figure 6 shows pixel output as a function of integration time for the hot pixels and normal pixels in light environments. Pixel output linearly increases with integration time below 0.12 s, and the slope of the curve is 5.8 × 10^5^ e^−^/s (corresponding to the photo-generated current). When the integration time is 0.2 s, the pixel output reaches full well capacity. Full well capacity is defined as the maximum amount of electrons stored in the pixel and is related to the light flux, VLOTG, and temperature [18]. The studied devices with 11 μm pixel pitch can reach the situation of full well capacity at a short integration time since BSI CIS feature high sensitivity. All the curves are the same for the selected hot pixels and normal pixels. This is because the photo-generated current is much larger than the dark current, and thus the influence of the dark current can be neglected.

**Figure 7:**
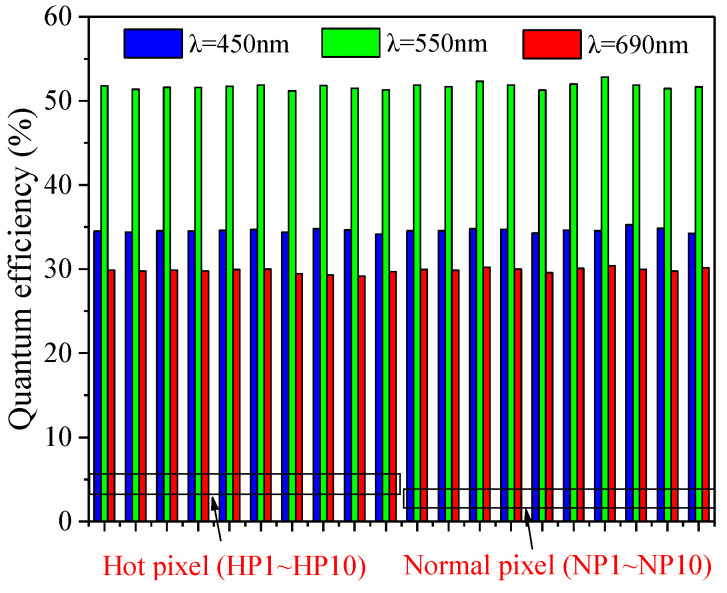
Quantum efficiency of the hot pixel and the normal pixel at the wavelength of 450, 550, and 690 nm.

QE represents the pixel’s ability to convert the incident photons to electrons at distinct wavelengths and is expressed as follows:

(1) $$\eta_{\left(\lambda \right)}=\frac{n_{e}}{n_{p}}*100\%$$

where *η*_(*λ*)_ is the QE, which is related to the wavelength, *n_e_* is the number of collected electrons, and *n_p_* is the number of impinging photons. The unit of pixel output is the digital number (DN) and can be converted into an electron (e^−^) through the CVG. CVG is defined as how much digital number variations are produced by an electron. Figure 7 depicts the QE of the hot pixels and normal pixels at the wavelength of 450, 550, and 690 nm. QE of the blue, green, and red lights are separately 34%, 51%, and 29%. Similarly, all the QE values for the hot pixels and normal pixels are almost the same. As mentioned above, QE is dependent on CVG. CVG can be tested using the mean-variance method, in which the slope of the linear region corresponds to CVG [19]. The measured CVG is 0.045 DN/e^−^ in the studied BSI CIS, and there is no difference in CVG for the hot pixels and normal pixels. Therefore, it can be concluded that radiation-induced hot pixels have no influence on the optoelectric performance in BSI CIS.

### 3.3. Pixel Performance in Dark Environments

**Figure 8:**
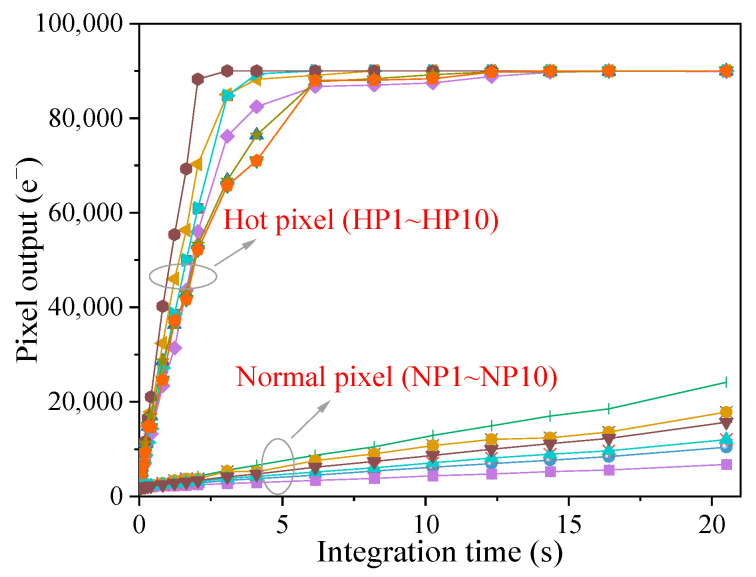
Pixel output as a function of integration time under dark environments.

Figure 8 shows pixel output as a function of integration time for the hot pixels and normal pixels in dark environments. For normal pixels, the pixel output linearly increases on the whole integration time (the slope of the curve is the dark current), and saturation is not observed. As for the hot pixels, the pixel output first exhibits a linear increase below 5 s and then reaches the situation of full well capacity at the integration time of 10 s. Above 10 s, the hot pixel has no response and is not an effective pixel anymore. This case should be concerned with an astronomical observation mission, in which the image sensors operate at a long integration time (up to several hours) to harvest information about remote stars. In addition, Ref. [20] has reported that the hot pixel is able to cause dark current blooming effects at the long integration time and seriously degrades image quality. However, no dark current blooming effect is observed in this work, even at the integration time of 60 s (the captured images are not shown here). When the pixel reaches the full well capacity, the excess electrons are spilled out of the PPD in two ways: (a) these electrons are transferred into the transfer gate channel through the thermal emission mechanism and are collected by the floating diffusion node. In our case, the transfer gate channel is in the accumulation regime, and the thermal emission probability of the electrons is small due to the presence of a high barrier. (b) the excess electrons are transferred in the epitaxial layer through the diffusion mechanism and are collected by the adjacent pixel. In this work, the studied devices have an 11 μm pixel pitch, and the diffusion length of dark electrons is smaller than the pixel pitch.

**Figure 9:**
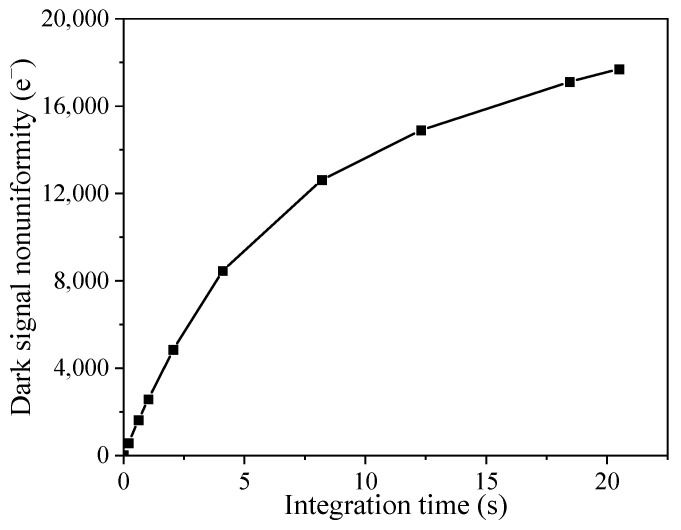
Dark signal nonuniformity versus integration time.

Dark signal nonuniformity refers to dark signal variations from pixel to pixel, and its unit is e^−^. Figure 9 shows the dark signal nonuniformity as a function of integration time. It can be seen that the dark signal nonuniformity linearly increases with integration time below 5 s and then gradually approaches saturation. This phenomenon is associated with the hot pixels since the hot pixel has an enhanced dark current compared to the normal pixels. When the integration time is smaller than 5 s, the number of hot pixels linearly increases with integration time. After 5 s, more and more pixels become hot pixels at the long integration time, and the dark signal difference between pixels slowly increases.

**Figure 10:**
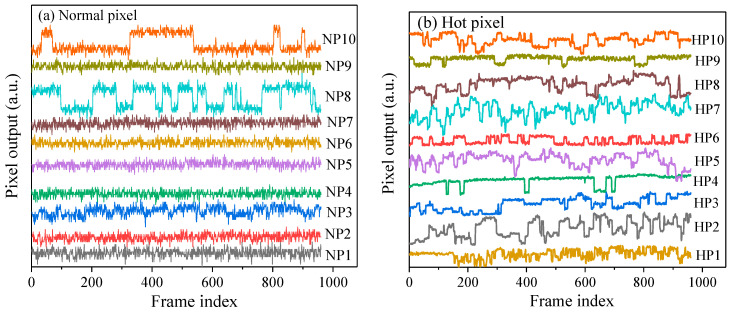
Pixel output versus time for the hot pixels and normal pixels.

Except for the hot pixel, the RTS pixel is another anomalous pixel in solid-state imaging devices and refers to dark current fluctuation between two or more discrete levels [21,22]. RTS pixel is usually encountered in space−borne imagers and gives rise to the calibration errors of the image sensors [6,21,23]. It is needed to point out that the RTS pixel is produced due to both displacement damage effects and total ionizing dose effects [24], while the hot pixel is only caused by displacement damage effects. To investigate the relationship between two kinds of anomalous pixels, the RTS pixel test was performed at 24 °C. Figure 10 depicts the pixel output versus the observation time for the hot pixels and normal pixels. For the sake of clarity, the unit of the pixel output is an arbitrary unit (a.u.). In the case of normal pixels, two normal pixels (corresponding to NP7 and NP10) exhibit RTS characteristics, and these two RTS pixels have two discrete levels. As for the hot pixels, all the hot pixels exhibit RTS fluctuations, and seven RTS pixels exhibit multi-discrete levels (more than three levels). The transition frequency of multi-level RTS pixels is larger than that of two-level RTS pixels. It can be known that there is a relationship between the hot pixels and the RTS pixels, which is the hot pixels are more likely to exhibit RTS characteristics, and these RTS pixels tend to show multi-levels. The most possible explanation responsible for the above phenomenon is that the RTS-related defects and hot-related defects have similarities, and the defect structure of the hot pixel is complex. On the one hand, the complex defect contains two or more configurations, and each configuration introduces a corresponding generation level within the Si bandgap. The stability of the complex defect is weak, and this defect can spontaneously switch from one configuration to another configuration, thus causing the random fluctuation of the pixel output with time. On the other hand, the complex defect introduces more energy levels compared to the defect with a simple structure, and thus there is a big probability of containing energy levels close to mid-gap. Thus, the complex defect gives rise to the production of hot pixels. Displacement damage produces isolated defects and cluster defects, and the difference between the two kinds of defects is defect density. Defect density is an important characteristic of defects and has a direct influence on the defect structure [10]. The cluster defect consists of amorphous regions in Si material and has a high defect density compared to the isolated defects. Hence, the cluster defect has a complex structure compared to the isolated defect and is the candidate resulting in the production of hot pixels.

To harvest the energy level of the defects, dark current was tested at distinct temperatures, and the corresponding activation energy was calculated according to the Arrhenius law [25,26,27].

(2) $$I_{dc}=Aexp\left(-\frac{E_{a}}{kT}\right)$$

where *I_dc_* is the dark current, *A* is the preexponential factor, *E_a_* is the activation energy, *k* is the Boltzmann constant, and *T* is the temperature. Considering the temperature dependence of each parameter [13], displacement damage-induced dark current can be described, as follows:

(3) Idc∝T2exp−Et−Ei+Eg2kT

where *E_t_* is the defect energy level, *E_i_* is the mid-gap energy level, and *E_g_* is the Si bandgap. Considering the temperature dependence of the Si bandgap, we obtain the expression of activation energy as a function of the energy level into the silicon bandgap as:

(4) $$E_{a}=0.649+\left|E_{t}-E_{i}\right|$$

**Figure 11:**
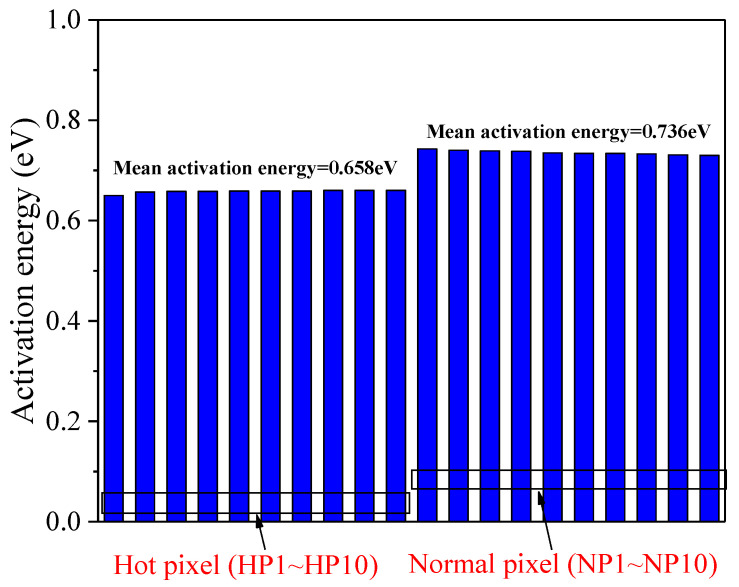
The dark current activation energy of the hot pixels and normal pixels at 24 °C.

It can be known from Formula (4) that the defect energy level is located at the mid-gap when the dark current activation energy is 0.649 eV, and the generation ability of the defect is maximized. Figure 11 reports the dark current activation energy of the hot pixels and normal pixels at 24 °C. It can be observed that the mean activation energy of hot pixels and normal pixels are 0.658 eV and 0.736 eV, respectively, which obeys the SRH theory. The result of activation energy suggests that the defect energy level of hot pixels is closer to mid-gap than the defects related to the normal pixels, and no EFE effect exists in the studied BSI CIS. Indeed, the EFE has been reported in CCD and CIS manufactured using an early fabrication process [11,25], and the studied BSI CIS is manufactured in a deep submicron process. 

## 4. Conclusions

In this paper, the effects of hot pixels on the pixel performance are investigated in pinned photodiode 0.18 μm backside illuminated CMOS image sensors irradiated by 10 MeV protons up to 39.4 TeV/g. After proton irradiation, hot pixels are produced by displacement damage through the elastic and inelastic nuclear scattering. Hot pixels are selected from the exponential tail of the dark current distribution, and normal pixels are randomly selected from the whole array. The method of selecting two kinds of pixels meets statistical requirements.

Hot pixel has no influence on optoelectric performance since the photo-generated current is much larger than the dark current, and the impact of the dark current can be neglected. However, it is worth pointing out that the effects of hot pixels on pixel performance in dark environments should be a concern. Hot pixel behaves as a “bright spot” in the captured dark images and gives rise to the increase of dark signal nonuniformity, which has an impact on the dim target detection mission. Dark signal nonuniformity degradation is strongly dependent on the number of hot pixels. A hot pixel is able to reach the situation of full well capacity at the long integration time without light illumination. Above the specific integration time, the hot pixel makes no response and is not an effective pixel anymore. This case has an impact on the astronomical observation mission, in which the observation at the long integration time is required for the image sensors. It is confirmed that there is a correlation between hot pixels and RTS pixels, which is the hot pixels tend to exhibit RTS characteristics. The possible explanation is that the defects responsible for the hot pixel have a complex structure. The complex defects not only spontaneously transition from one configuration to another configuration due to the instability of the defects, but also have a big probability of introducing energy levels close to mid-gap. According to the characteristics of displacement damage-induced defects, cluster defects, which have complex defect structures, are more likely to cause the production of hot pixels. Furthermore, the result of dark current activation energy suggests that radiation-induced hot pixel is caused by defects with energy levels close to mid-gap.

## Figures and Tables

**Table 1:** Information of hot pixels and normal pixels.

Hot Pixel	Dark Current (e^−^/s)	Location	Normal Pixel	Dark Current (e^−^/s)	Location
HP1	8549.5	[299, 276]	NP1	309.9	[155, 95]
HP2	8360.4	[119, 140]	NP2	347.3	[3, 81]
HP3	8254.9	[143, 127]	NP3	358.2	[483, 225]
HP4	8149.5	[143, 191]	NP4	375.8	[597, 138]
HP5	7936.3	[208, 151]	NP5	415.4	[63, 527]
HP6	7907.7	[271, 24]	NP6	433.0	[201, 47]
HP7	7696.7	[158, 86]	NP7	444.0	[334, 206]
HP8	7582.4	[288, 246]	NP8	461.5	[246, 189]
HP9	7575.8	[263, 218]	NP9	496.7	[634, 301]
HP10	7547.3	[129, 75]	NP10	512.1	[48, 190]

## Data Availability

Data is not available due to privacy restrictions.
